# S06-4: How is organised the exercise training of the outpatient phase III cardiac rehabilitation in the Greater Region?

**DOI:** 10.1093/eurpub/ckae114.225

**Published:** 2024-09-26

**Authors:** Alexis Lion, Patrick Feiereisen, Charles Delagardelle

**Affiliations:** Fédération Luxembourgeoise des Associations de Sport de Santé; Centre Hospitalier de Luxembourg; Fédération Luxembourgeoise des Associations de Sport de Santé

## Abstract

**Purpose:**

The phase III cardiac rehabilitation includes lifestyle modifications and exercise training. While the phase III cardiac rehabilitation is recommended, there is no clear recommendation regarding its organisation. To investigate how exercise training in phase III cardiac rehabilitation was organized, we conducted a pilot study in the Greater Region (an international region composed by territories from Belgium, France, Germany, and Luxembourg).

**Methods:**

We emailed and called five organisations offering or coordinating the exercise training of the phase III cardiac rehabilitation in the Greater Region and investigated different parameters: status of the contacted organisations, profile of their participants, recruitment process, organisation of the exercise training classes. We also screened the websites and registration forms of the different contacted organisations.

**Results:**

Non-profit organisations organised exercise training in phase III cardiac rehabilitation in all 4 territories of the Greater Region. The profile of the participants in the exercise training varied across the different territories from participants with cardiac diseases only to everybody. For the 1st registration, participants should provide a medical clearance or/and a referral letter from a medical doctor or a cardiologist. In some territories, a stress test was additionally required for participants with cardiac diseases and participants presenting risk factors for cardiovascular disease. Annual medical clearance was not mandatory in some territories. In two territories, participants may be referred into a starter programme at the beginning of phase III cardiac rehabilitation. After this starter programme, participants were invited to continue into the regular programme. In most cases, exercise training was supervised by physiotherapists or sports therapists. The medical surveillance of the exercise training by a medical doctor and/or a nurse was often recommended but rarely and heterogeneously implemented.

**Conclusions:**

The exercise training of the outpatient phase III cardiac rehabilitation is organised differently in the 4 territories of the Greater Region. Variation exists in implementation of referrals, medical clearance, and medical surveillance. Guidelines based on the evidence and the good practice should be elaborated. It will help non-profit organisations to better organise and administrate exercise training of the phase III cardiac rehabilitation.

